# Analysis of regulatory effect of miR-149-5p on Sphingosine-1-phosphate receptor 2 of pericytes and its neuroprotective molecular mechanism after acute cerebral ischemia reperfusion in rats

**DOI:** 10.1080/21655979.2021.1947167

**Published:** 2021-07-05

**Authors:** Zhenxing Yan, Yiting Deng, Yang Zou, Siqin Liu, Kaifeng Li, Juan Yang, Xihua Guo, Rongni He, Wenxia Zheng, Huifang Xie

**Affiliations:** a Department of Neurology, Zhujiang Hospital, Southern Medical University, Guangzhou, PR China; b Second Clinical College, Southern Medical University, Guangzhou, PR China; c Second School of Clinical Medicine, Southern Medical University, Guangzhou, PR China; d Shenzhen Hospital, Southern Medical University, Guangzhou, PR China; e Department of Neurology, Shunde Hospital, Southern Medical University, Foshan, PR China

**Keywords:** Mir-149-5p, acute cerebral ischemia reperfusion, pericytes, S1PR2, neuroprotection, mmp9 protein

## Abstract

To investigate the effect of miR-149-5p on sphingosine-1-phosphate receptor 2 (S1PR2) expression level and contents of matrix metalloproteinase (MMP-9) and superoxide dismutase (SOD) in the pericytes after acute cerebral ischemia reperfusion in rats, so as to clarify the neuroprotective molecular mechanism induced by miR-149-5p and provide references for the treatment of neurological diseases, 60 male SD rats aged 7–8 weeks were selected and divided randomly into test group (establishing middle cerebral artery occlusion (MCAO) model) and control group (no modeling). Rat pericytes and peripheral cerebral infarction tissues were collected 12 h, 1 d, 3 d, 5 d, and 7 d after MCAO modeling, respectively. The pericytes were identified by immunofluorescence assay (IFA) and transfected with miR-149-5p. Fluorescence quantitative PCR (FQPCR) and Western blot were adopted to detect S1PR2 expression level. The expression of S1PR2 in MCAO model rats was detected by IFA. Immunohistochemistry (IHC) and quantitative real-time PCR (qRT-PCR) were used to detect the changes of MMP9 protein and mRNA levels of SOD1, SOD2, and SOD3 in brain tissue. The results showed that mRNA level and protein expression level of S1PR2 in the test group were higher than those in the control group three days after MCAO modeling (*P* < 0.05); the expression of S1PR2 increased 12 h after MCAO modeling and returned to the normal level on the 5th day, and the content of MMP9 protein in brain tissue of the test group was significantly lower than that of the control group (*P* < 0.05); the mRNA levels and SODs activity of SOD1, SOD2, and SOD3 in the test group were higher than those in the control group (*P* < 0.05). Therefore, miR-149-5p played a neuroprotective role by regulating S1PR2 to change the expression levels of SODS and MMP9.

## Graphical Abstract

**Figure:**
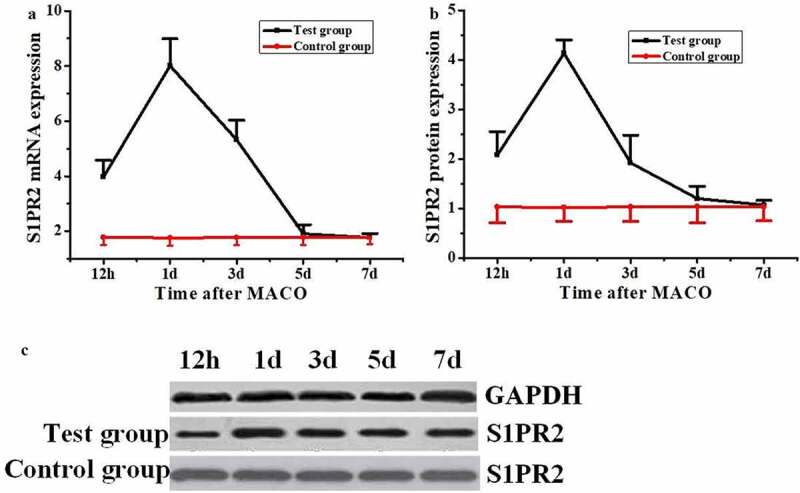

## 1. Introduction

Ischemic stroke is a sudden and acute disorder of cerebral blood circulation, which is one of the diseases with high morbidity, mortality, and recurrence rate at present [1]. Ischemic stroke is the first cause of disability and death in China, which brings huge economic burden to society and family. Early reperfusion established by recombinant tissue-type plasminogen activator thrombolysis and/or mechanical recanalization is the most clinically proven therapy. However, the blood-brain barrier (BBB) is damaged in the treatment of cerebral apoplexy with acute cerebral ischemia/reperfusion, which eventually leads to brain edema and brain tissue damage and other symptoms [2]. BBB is the basic neural unit for the interaction of microvascular endothelial cells in the brain with other cells. When the protein expression related to BBB integrity changes due to external stimuli and changes in the body, BBB function disorders will occur, leading to various complications [3].

The endothelial part of BBB is the main site of reactive oxygen species (ROS) production. Current studies found that inflammatory mediators, vascular endothelial growth factors, and micro-RNAs (miRNAs) all had certain effects on BBB [4]. Studies also pointed out that miRNA was a key regulatory molecule of ischemic stroke disease and was also involved in the process of BBB destruction after stroke [5]. MiR-149 plays an important role in the development and progression of coronary artery disease, tumors, and inflammation. Recent studies showed that miR-149 polymorphism was associated with the incidence of ischemic stroke. Studies also pointed out that the expression of miR-149-5p in the cells of stroke model rats was significantly reduced, which was negatively correlated with the expression of S1PR2 [6]. Sphingosine-1-phosphate receptors (SIPRs) bind to Sphingosine-1-phosphate (S1p) and participate in the regulation of cell proliferation, inflammation, and cell migration [7]. S1PR2 gene can block the occurrence and development of cerebral edema and other complications after stroke through the regulation of matrix metalloproteinase (MMP-9) [8]. S1PR2 is also associated with oxidative stress, mediates ROS regulation of BBB permeability, and mimics reactive oxygen species in mouse ischemia/reperfusion (I/R) tissue. ROS levels increase with the degree of ischemia. Studies revealed that increased ROS was an important cause of reperfusion injury, and its content was regulated by SOD1, SOD2, and SOD3. When the amount of SODS synthesis increases, the ROS content will be significantly reduced, thus alleviating the I/R injury and achieving the neuroprotective effect [9]. However, the role of S1PR2 in pericyte ischemia response remains unknown. Based on the above research results, it was speculated that miR-149-5p may affect the changes of ROS and SODS content through the regulation of S1PR2, and may be involved in the neuroprotective mechanism.

Based on the above speculation, the rat model of acute ischemia reperfusion was established to investigate the effects of miR-149-5p on the expression levels of S1PR2 and SODS at the cellular and molecular levels. This work aimed to analyze the regulatory effect of miR-149-5p on pericytes S1PR2 after acute ischemia reperfusion in rats and whether it was involved in the neuroprotective mechanism, so as to provide reference basis for the treatment of late nervous system diseases.

## 2. Materials and methods

### 2.1 Experimental animals and grouping

Sixty healthy male SD rats aged 7–8 weeks, weighing 265–305 g were selected, with 5 in each cage in SPF laboratory. The room temperature was 25°C, and relative humidity was about 55%. They were given free drinking water and diet under 12 h of light. After 2 weeks of adaptive feeding, all rats were randomly divided into two groups: test group (30 rats) and control group (30 rats). I/R model was established in the test group and not in the control group. All animal experiments in this study were approved by the experimental animal control committee, and the experimental methods were conducted in accordance with the approval guidelines.

### 2.2 Construction of acute cerebral I/R model in rats

The middle cerebral artery occlusion (MCAO) model in rats was established by reference to the improved line occlusion method and modified based on that [10]. Rats were anesthetized by intraperitoneal injection of 10% chloral hydrate before the surgical procedure. After satisfactory anesthesia, the rats were supine fixed on the operating table, neck hair was clipped, and skin was disinfected with iodine volt. The rectal temperature of rats was maintained at 37 ± 1°C using heating pads. The right common carotid artery of rats was fully exposed by blunt separation under a microscope. The internal carotid artery and external carotid artery were separated. The distal end of the isolated external carotid artery was ligated with a poly-L-lysine-coated 4–0 monofilament nylon thread with a length of 11 mm, and the spare suture was placed into the external carotid artery. The common carotid artery and the distal end of the internal carotid artery were clamped to block the blood flow of the middle cerebral artery. A small opening was cut near the heart end of the ligation of the external carotid artery with a Vannas spring shears to introduce the suppository thread, and the suppository thread was tied to the vessel with the prepared suture. The embolus passed through the common carotid artery and entered the internal carotid artery, which was divided into two small arteries when entering the brain, one of which was the pterygopalatine artery. When the embolus entered, the pterygopalatine artery should be gently pinched with forceps to avoid the embolus from entering the pterygopalatine artery. When the thread entered, it should be stopped in time when it encountered resistance, and it was tied tightly with sutures to ensure that the thread cannot move. The skin was disinfected and sutured with iodophor cotton ball after suture. After the nylon thread was placed for 2 h, it was removed and then perfusion was performed. After no bleeding was confirmed, penicillin was put into the surgical site and the skin was sutured. The cerebral blood flow (CBF) in the ischemic area of rats was monitored by the CBF detector of BD Company during operation. After the postoperative rats woke up, 2-mL penicillin injection was injected into the hind legs of the rats to prevent infection.

### 2.3 Isolation and treatment of pericytes in the cerebral I/R model rats

Three rats were sacrificed 12 h, 1 d, 3 d, 5 d, and 7 d after the establishment of the I/R model, and pericytes and brain tissue of all rats were collected. Rat pericytes were obtained by referring to the previous method [11] and modified accordingly. Rats were anesthetized with 10% chloral hydrate before the brain tissues were collected. Brain stem, white matter, and surface blood vessels were carefully removed. The cerebral cortex tissue was cut into pieces and put into the pre-cooled PBS to obtain homogenate, which was centrifuged at 500 rpm for 5 min in a low-temperature centrifuge to collect precipitation. It was suspended in 20% Percoll and centrifuged at 500 rpm for 20 min, and was incubated with 1000 U/mL DNase I and 0.1% collagenase II at 37°C for 1 h. The precipitation was collected after the centrifugation and suspended in 20% Percoll, then the centrifugation was performed at 4°C for 15 min, after which the middle layer would be collected. It was resuspended in a dual-resistance (penicillin + streptomycin) endothelial medium containing pericyte growth factor, and cultured in a constant temperature incubator (37°C, 5%CO_2_), the liquid was changed once for 3 days. The cultured pericytes were identified by IFA. The test group pericytes Lipofectamine 2000 were transfected with miR-149-5p at 50 nmol/L (Thermo, USA). Transfection was performed according to the instructions of the transfection kit. The cells were placed in 96-well plate one day before transfection, and the cell density was between 40% and 60%. After the cells were adherent to the wall, serum-free medium was added to culture them overnight at 37°C, and the cell density at transfection was 60–70%.

### 2.4 Quantitative real-time PCR (qRT-PCR) detection assay

After the rats were killed, the brain tissues were stripped off and washed with normal saline precooled at low temperature, and were put into a homogenizer and cut into pieces as far as possible. The total RNA of pericytes or brain tissue was extracted by TRIzol method, and the extracted total RNA was detected by agar-gel electrophoresis. Hifair® III 1st Strand cDNA Synthesis SuperMix for qPCR retroviruses kit (Takara) was adopted for reverse transcription. The reverse transcription was performed at 37°C for 15 min and 85°C for 5 s. Real-time FQPCR primer for synthesis of S1PR2 gene was S1-F: 5ʹ-AAATCCAATACGGTACAAACG-3ʹ; S1-R: 5ʹ-GGTCA GACAGCA CCCACA-3ʹ. Real-time FQPCR primers for synthesis of SOD1 gene was SO1-F: 5ʹ-GAGACCTG GGCAATGTGACT-3ʹ; SO1-R: 5ʹ-GTTTACTGCGCAATCCCAAT-3ʹ. Primer for synthesis of SOD2 was SO2-F: 5ʹ – CCGAGGAGAAGTACCACGAG-3ʹ; SO2-R: 5ʹ- GCTTGATAGCC TCCAGCAAC-3ʹ. Primer for synthesis of SOD3 was SO3-F: 5ʹ- ATCCCACAAGCCCCTAGTCT-3ʹ; SO3-R: 5ʹ- GTGCTATGGGGACAGGAAGA-3ʹ. GAPDH was taken as internal reference, primer of GAPDH gene was Fm: AGTTCAACGGCACAGTCAAGG; Rm: CAGCCTTCTCCATGGTGGTG. Each sample was amplified with the above three pairs of primers, and each reaction was repeated for three times.

The qRT-PCR reaction system is as follows: 1 μL cDNA, 0.3 μL SYBR Green, 10 μL 2× Taq PCR Master Mix, 0.5 μL forward primer, and 0.5 μL reverse primer. Then, ddH_2_O was added to make up the total volume to 20 μL. The amplification conditions of the PCR reaction were as follows: 94°C for 5 min; 94°C for 10 s, 60°C for 20 s; 72°C for 30 s; with a total of 40 cycles; then 72°C for 2.5 min, and 40°C for 2.5 min. The reaction was carried out in Exicycler TM 96 fluorescent quantitative PCR instrument. The 2^−ΔΔct^ method was used to calculate the relative expression level [12].

### 2.5 The protein expression of S1PR2 gene detected by Western blot

The peripheral infarcted tissue after acute ischemia reperfusion was put into a homogenizer and cut into pieces as much as possible. 1 mL of pre-configured lysate (RIPA protein lysate: protease inhibitor PMSF = 1:100) was added for grinding, so that the tissue was fully lysed to extract the total protein. The total protein was determined by bicinchoninic acid (BCA) assay. 12% Tris-glycine gradient gel was used to separate cell proteins by sodium dodecyl sulfate polyacrylamide gel electrophoresis (SDS-PAGE) at 80 V−120 V. The protein was transferred to the polyvinylidene fluoride (PVDF) membrane by the wet transfer method at 4°C and 80 V. Then, it was sealed with 5% skimmed milk at room temperature for 1 h. Protein was transferred to polyvinylidene fluoride (PVDF) membrane by wet transfer method, blocked by 5% skimmed milk, and incubated with antibodies of S1PR2 and GAPDH diluted by 1:1000 at 4°C overnight. Protein was washed thoroughly with tris-buffered saline tween (TBST) for three times, 10 min for each time, and incubated with horseradish peroxidase-coupled secondary antibody at room temperature for 2 h; TBST was used to wash it thoroughly for three times, 10 min for each time, and color developing solution was added. The image of protein was exposed automatically on the developer, and the grayscale scan was conducted. The protein expression was analyzed according to the gray value.

### 2.6 Detection of MMP9 protein in rat brain tissue through immunohistochemical assay

Immunohistochemical assay was used to detect the protein content of MMP9 in the brain tissue of the test group and the control group, and anti-MMP9 antibody was taken as the primary antibody. Immunohistochemical operations were carried out in accordance with routine operations [10]. The expression of MMP9 in rat brain tissue was observed under the light microscope. Staining of cytoplasm and nucleus with brown or tan was deemed as positive, while staining of cytoplasm and nucleus with blue was negative. Image-Pro Plus 6.0 was adopted to determine the average optical density value of the positive substance.

### 2.7 Immunofluorescence assay

Rats were sacrificed 12 h, 1 d, 3 d, 5 d, and 7 d after I/R model was established, respectively. Perfusion was performed with precooled PBS and precooled 4% paraformaldehyde in turn. Brain tissue and pericytes were collected. The brain tissue was dehydrated and sectioned (20 μm), 5% bovine serum (with 0.3% Triton X-100) was added for sealing for 1 h after washing with PBS for three times, and incubated with primary antibody overnight at low temperature. Washed with PBS for three times, the tissue was added with fluorescently labeled secondary antibody and incubated at room temperature for 2 h. 4ʹ, 6-diamidino-2-phenylindole (DAPI) staining was performed for 10 min, and the sections were sealed. Photographs of the tissue were taken with the laser scanning confocal microscope.

### 2.8 Detection of SODs enzyme activity in rat brain tissue

After the rats were killed, their brain tissue was taken out and the pre-cooled 50 mmol/L Tris-HCl (pH = 7.0) was added into the homogenizer for tissue homogenization. After centrifugation, supernatant was taken and SODs enzyme activity in brain tissue was detected by the SODs kit (Wako Company, USA). Specific operation steps were carried out in accordance with the instructions.

### 2.9 Statistical analysis

SPSS 19.0 was adopted for data statistics and analysis. Measurement data were expressed as mean plus or minus standard deviation ($\overset{ˉ}{X}$±s) and count data as percentage. The *t*-test was used for measurement data subjected to normal distribution, Wilcoxon test was used for measurement data subject to normal distribution, and *P* < 0.05 was considered statistically significant.

## 3. Results

### 3.1 Main works of this research

**Figure 1.**
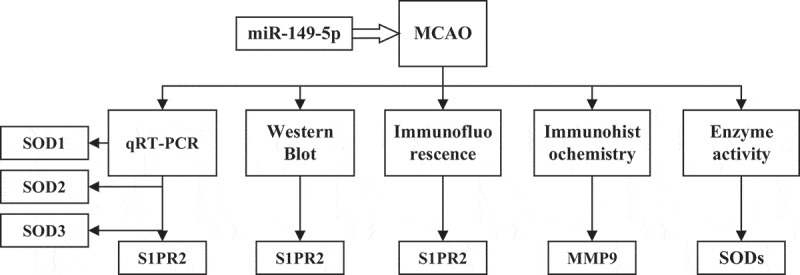
Frame diagram of the main contents of this study

The purpose of this study was investigating the effects of miR-149-5p on the contents of SIPRS2, matrix metalloproteinase (MMP-9), and SODs in pericytes after acute cerebral ischemia and reperfusion in rats, so as to clarify the neuroprotective molecular mechanism induced by miR-149-5p. In this study, a middle cerebral artery occlusion (MCAO) model was established in healthy male SD rats. Rat pericytes were collected and transfected with miR-149-5. Then, fluorescence quantitative PCR was used to analyze the changes of S1PR2, SOD1, SOD2, and SOD3 mRNA levels. Western blot and immunofluorescence were used to detect the protein expression of S1PR2. In addition, immunohistochemistry was used to detect the changes of MMP9 protein levels in brain tissues, and the activity of SODS enzyme was detected (Figure 1).

### 3.2 Immunofluorescence assay to identify primary BMPs cultured in vitro in rats with acute I/R

**Figure 2.**
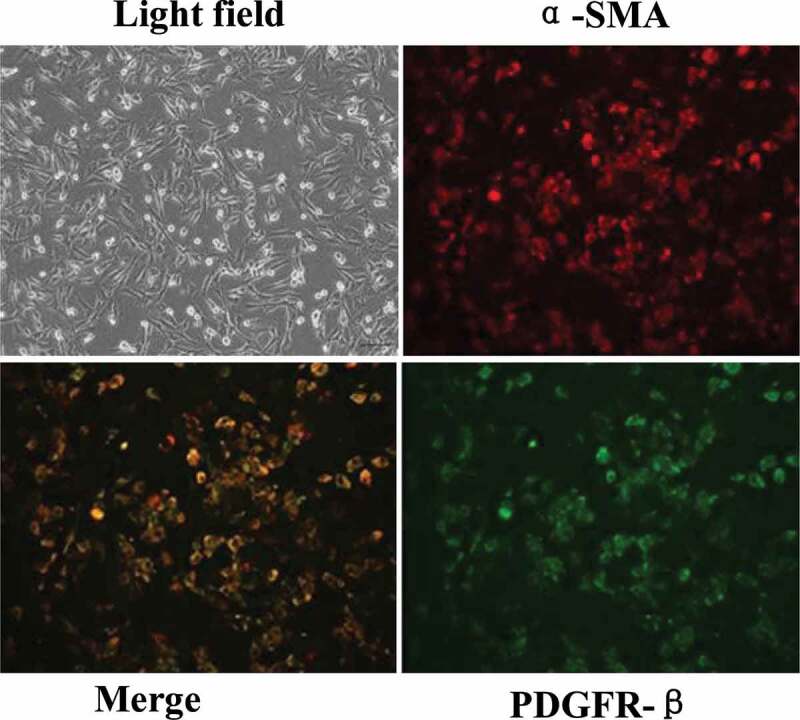
Primary BMPs identified by immunofluorescence co-staining of α-SMA and PDGFR-β

The isolated rat brain microvascular pericytes (BMPs) were cultured in vitro. The pericytes were found to be radially or elliptically arranged during culture. With the increase of cell-culture algebra, positive platelet-derived growth factor receptor-β (PDGFR-β) increased, other cells such as neurons and microglia cells were fewer and fewer. After three times of subculture, almost no other cells were found, indicating that pericytes could be purified by subculture. The identification results of the third generation after subculture by α-smooth muscle actin (α-SMA) and PDGFR-β were shown in Figure 2. After the third generation, α-SMA and PDGFR-β were double positive, indicating that the purified perivascular cells had been obtained.

### 3.3 Detection of S1PR2 expression in BMPs of acute I/R rats

**Figure 3.**
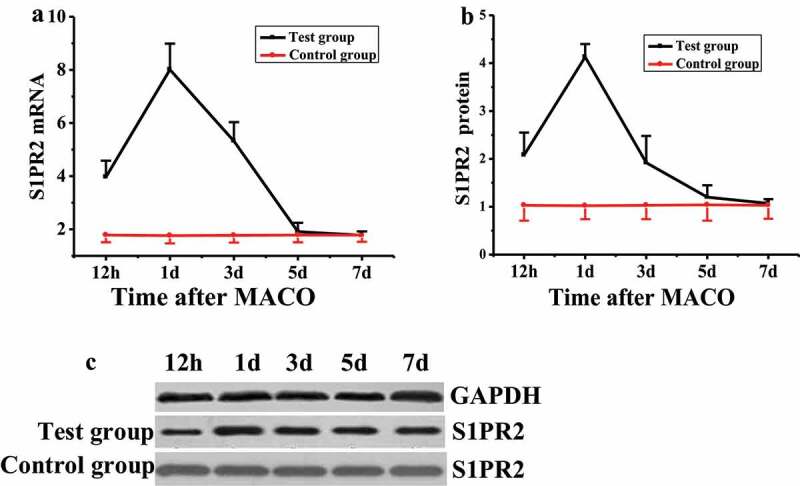
Changes of S1PR2 expression in pericytes of MCAO model rats

The expression level of S1PR2 in pericytes of rats after acute I/R was detected by quantitative reverse-transcription PCR (qRT-PCR) and Western blot, respectively. The results were shown in Figure 3. The mRNA level of S1PR2 in rats in the control group remained unchanged during the test, while that the test group was significantly higher than that of the control group 12 h after MCAO modeling (Figure 2a), with significant difference (*P* < 0.05). The mRNA level of S1PR2 reached the highest value (8.01 ± 0.98) on the first day after MCAO modeling, showed a downward trend on the third day, and decreased to the lowest level on the fifth and seventh days, with no significant difference compared with the control group (*P* > 0.05). The S1PR2 protein expression level of rats in the control group remained unchanged during the test, while that of the test group was significantly higher than that of the control group at 12 h after MCAO modeling (Figure 2b and Figure 2c), with a significant difference (*P* < 0.05). On the first day after MCAO modeling, the expression of S1PR2 protein reached the highest value (4.13 ± 0.27), which was significantly different from that of the control group (*P* < 0.05). The expression of S1PR2 protein decreased to the lowest level on the seventh day after MCAO modeling, with no significant difference compared with the control group (*P* > 0.05).

(A: changes of S1PR2 mRNA level in pericytes detected by q RT-PCR; B: protein expression of S1PR2 detected by Western blot; C: image of S1PR2 protein expression.)

### 3.4 Immunofluorescence detection of S1PR2 expression

**Figure 4.**
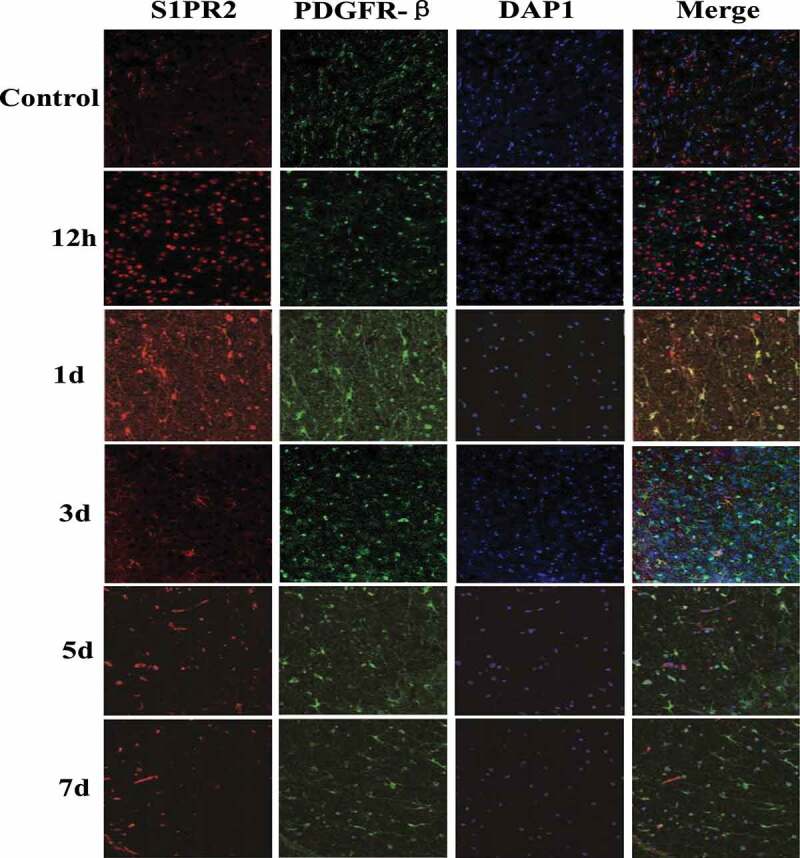
Changes of S1PR2 expression in pericytes of control group and test group at different time points after MCAO modeling

Immunofluorescence staining was adopted to detect the expression of S1PR2 in peripheral infarcted tissues of MCAO rats. And PDGFR-β was taken as the pericytes marker, and the results were shown in Figure 4. There was almost no S1PR2 expression in pericytes of control group rats, and the expression of S1PR2 in rat pericytes increased significantly from 12 h after MCAO modeling, which reached the maximum value on the first day after MCAO modeling and returned to normal level on the fifth day after MCAO modeling.

### 3.5 Detection of MMP9 protein content in rat brain tissue

**Figure 5.**
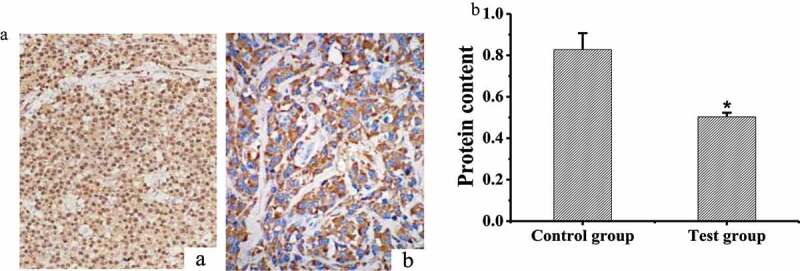
Detection of MMP9 protein content

IHC was adopted to stain the MMP9 protein in the brain tissues of the two groups of rats, and the results were shown in Figure 5a that MMP9 protein was mainly expressed on the cell membrane. The content of MMP9 protein in the brain tissues of the two groups of rats was measured and compared, and the results were shown in Figure 5b. The protein content of MMP9 in rat brain of test group was (0.503 ± 0.02) and that in the control group was (0.827 ± 0.08). The content of MMP9 protein in the test group was significantly lower than that in the control group (*P* < 0.05).

(A: staining results of MMP9 protein expression in rat brain tissue, a: the control group; b: the test group; B: comparison of MMP9 protein content in brain tissue between the two groups, * indicated significant difference compared with the control group, *P* < 0.05)

### 3.6 Detection of mRNA levels of SOD1, SOD2, and SOD3 in rat brain tissue

**Figure 6.**
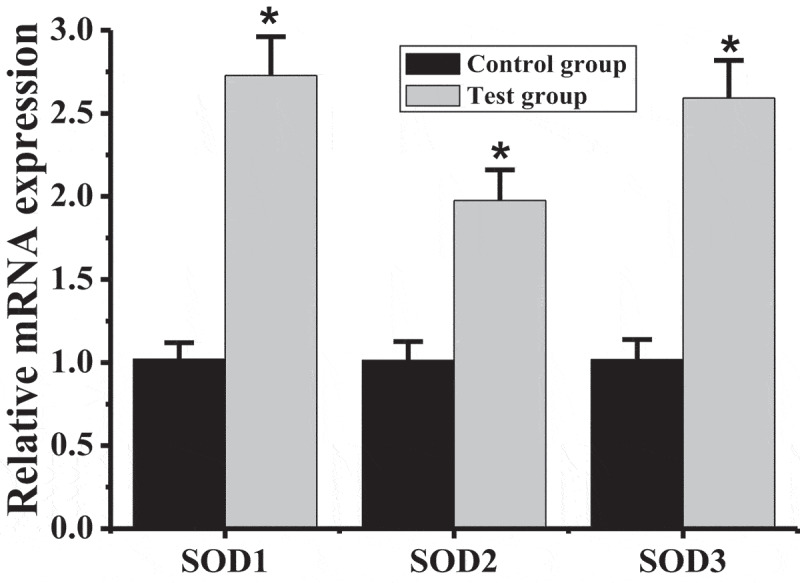
Comparison of SODs mRNA levels between test group and control group

The mRNA levels of SOD1, SOD2, and SOD3 in the brain tissues of rats in the test group and the control group were detected, and the results are shown in Figure 6 that the mRNA levels of SOD1, SOD2, and SOD3 in rats in the test group were all higher than those in the control group, with significant differences (*P* < 0.05).

(* indicated significant difference compared with the control group, *P* < 0.05)

### 3.7 Detection of SODs enzyme activity in rat brain tissue

**Figure 7.**
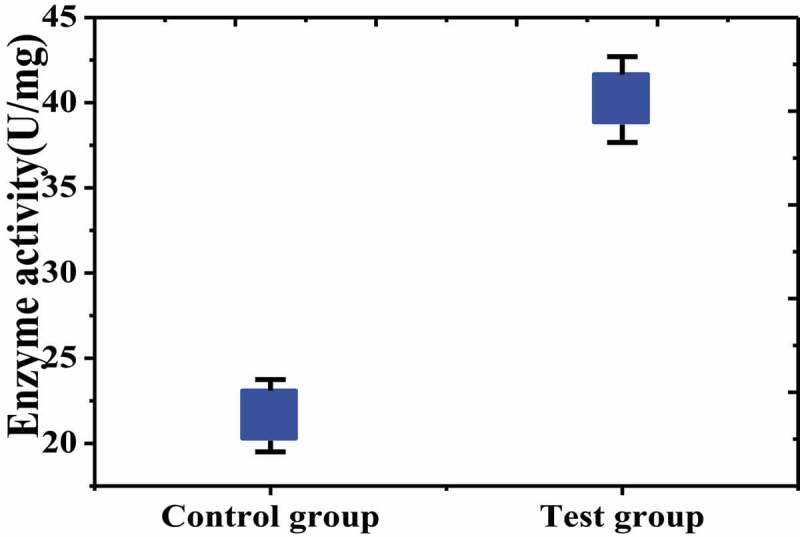
Comparison of SODs enzyme activity between the two groups

The SODs enzyme activity in the brain tissue of the test group and the control group was detected and compared. The results were shown in Figure 7 that the SODs enzyme activity in the brain tissue of the test group was (40.18 ± 1.517) U/mg, while that in the control group was (21.62 ± 1.123) U/mg, which was significantly lower than in the test group, and there was a significant difference between the two groups (*P* < 0.05).

## 4. Discussion

After acute ischemic reperfusion, BBB would be damaged in certain degrees, leading to intracranial hemorrhage, cerebral edema, and other complications [13]. A large number of studies showed that BMPs played an important role in the integrity of BBB [14]. S1P and S1PRs were involved in cell adhesion, migration, and BBB permeability changes [15]. In this study, it was found that the expression level of S1PR2 in pericytes of rats transfected with miR-149-5p showed a trend of increasing firstly and then decreasing after acute ischemia perfusion. The mRNA and protein levels of S1PR2 in the control group remained unchanged during the test. The mRNA level and protein expression level of S1PR2 in the test group reached the highest value (8.01 ± 0.98) on the first day after MCAO modeling, which was significantly different from that of the control group. The expression of S1PR2 protein decreased to the lowest level on the 7th day after MCAO modeling, with no significant difference compared with the control group (*P* > 0.05), which was consistent with the research of Matsumoto et al. (2020) [16], who deemed that the decreased S1P level was significantly correlated with the death of nerve cells in ischemic stroke. S1PR2 could activate the cytoskeleton contraction mechanism to play a role in increasing capillary permeability, and S1PR2 expression would significantly increase in endothelial cells after ischemic stroke, leading to neurovascular injury [17]. The results of this study showed that S1PR2 expression was significantly increased in pericytes of rats with ischemia and reperfusion. Wu et al. (2020) [18] found that after ischemic stroke, the up-regulated S1PR2 expression in brain microvascular activated MMP9 and increased brain microvascular permeability. And studies showed that miR-149-5p could reduce the body’s inflammatory response by regulating the MMP9 expression. MMP9 destroyed the integrity of BBB, thereby causing cerebral hemorrhage and cerebral edema, and had certain toxic and side effects on neurons [19]. The results of this study showed that the content of MMP9 protein in the brain tissue of the test group was significantly lower than that of the control group (*P* < 0.05), which was consistent with the current results, indicating that miR-149-5p had a certain effect on the expression of MMP9 protein.

The change of SOD expression in the brain had different degrees of influence on the local ROS level in brain. Studies showed that after activation, SODs played a protective role on cerebral nerves, enhanced SODs activity, and caused a significant decrease in ROS content [20]. Some studies pointed out that SOD1 and SOD3 are very important for limiting O^2-^ accumulation in cerebral vessels. Mice lacking the SOD1 gene had elevated O^2-^ levels and impaired cerebrovascular diastolic function. Excessive destruction of O^2-^ by SODs can increase the generation of dismutase products and powerful cerebrovascular diastolic H_2_O_2_ [21]. The results of this study showed that the mRNA levels of SOD1, SOD2, and SOD3 in the test group were all higher than those in the control group, with significant differences (*P* < 0.05); the activity of SODs enzyme in test group was significantly higher than that in control group (*P* < 0.05), which were consistent with the current studies that increased SODs expression in brain tissue could significantly reduce nerve injury. The results of this study indicated that enhanced SODs activity could reduce ROS levels in rats after ischemia reperfusion and played a protective role on nerves.

## 5. Conclusion

In this study, a rat model of acute cerebral ischemia reperfusion was established, and miR-149-5p was infected with pericytes in the test model group, so as to detect the regulatory and neuroprotective effects of miR-149-5p on the expression level of S1PR2 in pericytes. The results showed that miR-149-5p significantly increased the expression of S1PR2, SOD1, SOD2, and SOD3, and significantly decreased the expression of MMP9 in pericytes after ischemia reperfusion, which alleviated BBB permeability and played a protective role on nerves. However, there were still some deficiencies in this study that only the regulation of miR-149-5p on S1PR2 in pericytes had been studied, while the specific pathways of the two had not been discovered and it was not clear whether miR-149-5p acted directly on S1PR2. In the later study, the regulation pathways of miR-149-5p on S1PR2 would be analyzed. In conclusion, miR-149-5p had a regulatory effect on S1PR2 of pericytes after acute cerebral ischemia reperfusion in rats and had a neuroprotective effect by regulating the expression levels of MMP9 and SODs.

## Research highlights

- miR-149-5p significantly up-regulated S1PR2 and SODs and down-regulated MMP9 in pericytes after ischemia-reperfusion.
- The effect of miR-149-5p on SIPRS2, MMP-9, and SODs in pericytes was analyzed based on rat MACO model, to clarify the mechanism of neuroprotection induced by miR-149-5p.
- miR-149-5p protected nerves by regulating change-related genes in pericytes after ischemia-reperfusion.
